# Complete Genome Sequence of Athalassotoga saccharophila Strain NAS-01, a Deep-Branching Thermophilic Lineage in the Phylum *Thermotogae*

**DOI:** 10.1128/MRA.00322-20

**Published:** 2020-04-16

**Authors:** Shingo Kato, Takashi Itoh, Moriya Ohkuma

**Affiliations:** a Japan Collection of Microorganisms, RIKEN BioResource Research Center, Tsukuba, Ibaraki, Japan; University of Delaware

## Abstract

Athalassotoga saccharophila strain NAS-01 (=JCM 19762^T^) is an anaerobic thermoacidophilic bacterium which is one of the deepest branching lineages of the phylum *Thermotogae*. Here, we report the complete genome sequence (1.96 Mbp) and two plasmid sequences (43.7 kbp and 43.5 kbp) of this strain.

## ANNOUNCEMENT

Diverse thermophiles thrive in terrestrial hot springs. The thermoacidophilic bacterium Athalassotoga saccharophila strain NAS-01 was isolated from a terrestrial hot spring in Japan, and it belongs to the phylum *Thermotogae* (1). The order *Mesoaciditogales*, including *A. saccharophila* and Mesoaciditoga lauensis (2), is the deepest branching clade in the *Thermotogae*. Previous genome analyses indicated that the chimeric feature of genomes of the order *Thermotogales* are due to horizontal gene transfer from members of the phylum *Firmicutes* and even the domain *Archaea* (3, 4). Although the draft genome sequence of *M. lauensis* has been publicly released (Integrated Microbial Genomes [IMG] identification no. 2579779163), none of the complete genome sequences of members of the *Mesoaciditogales* have been reported so far.

Here, we report the complete genome sequence of *A. saccharophila* strain NAS-01. This strain was cultivated in an Fe(III)-containing medium (JCM medium no. 1014) under anaerobic conditions at 55°C and pH 6.0 as previously described (1). Total DNA was extracted from cells using a DNeasy UltraClean microbial kit (Qiagen, Germany) and purified as previously described (1). The purified DNA was applied to shotgun DNA sequencing using a MiSeq instrument (Illumina, USA) with a QIAseq FX DNA library kit (Qiagen), a MiSeq reagent kit version 3 (600 cycles, 300-bp paired-end reads; Illumina), and a MinION device (Oxford Nanopore Technologies [ONT], United Kingdom) with a flow cell (FLO-MIN107 R9 version; ONT) and an R9.5 1D^2^ sequencing kit (ONT). The MiSeq and MinION sequencing runs resulted in 4,325,178 reads with an average read length of 220 bases and 3,859,209 reads with an average read length of 1,051 bases, respectively. The MiSeq reads were quality trimmed and filtered using the read quality control pipeline of MetaWRAP version 1.0.5 (5) with the default settings. The low-quality MinION reads (<8 read quality score and <5,000 bases long) were filtered using NanoFilt version 2.2.0 (6). The trimming and filtering of the reads from the MiSeq and MinION runs resulted in 4,310,774 reads with an average read length of 217 bases and 14,710 reads with an average read length of 8,616 bases, respectively. The high-quality short and long sequences were coassembled using Unicycler version 0.4.7 (7) with the conservative mode, resulting in three circular contigs (1,957,762, 43,703, and 43,534 bp with G+C contents of 40.64%, 35.97%, and 35.16%, respectively). The gene prediction and annotation were performed using the Rapid Annotations using Subsystems Technology (RAST) server version 2.0 (8), Prokka version 1.13 (9), and the DDBJ Fast Annotation and Submission Tool (DFAST) (10) with the default settings. The annotation was manually curated based on the results from the Kyoto Encyclopedia of Genes and Genomes (KEGG) (11), InterProScan version 5.33-72.0 (12), and eggNOG version 4.5.1 (13).

The longest circular contig corresponding to the chromosomal DNA contained 1,914 protein-coding regions (CDSs), 2 copies of rRNA gene operons (5S, 16S, and 23S), and 48 coding regions of tRNAs. CDSs for the complete set of the Embden-Meyerhof-Parnas pathway and those for the pentose phosphate pathway were found. In contrast, few CDSs involved in the tricarboxylic acid (TCA) cycle were found. Remarkably, the genome encodes CDSs for both F-type and V-type ATPases. The other two circular contigs, defined as pATS1 (43.7 kbp) and pATS2 (43.5 kbp), correspond to plasmid DNAs. pATS1 and pATS2 contained 43 and 47 CDSs, respectively. Further genome sequencing and analysis of members of the deepest clade, *Mesoaciditogales*, will provide insights into the unique evolutionary history of the phylum *Thermotogae*.

### Data availability.

The sequences determined in the present study have been deposited in DDBJ/ENA/GenBank under the accession no. AP019551 for chromosome DNA, AP019552 for pATS1, and AP019553 for pATS2. The raw sequence data have been deposited under the accession no. DRA008891 for the MiSeq run and no. DRA009879 for the MinION run.

## References

[1] Itoh T, Onishi M, Kato S, Iino T, Sakamoto M, Kudo T, Takashina T, Ohkuma M 2016 *Athalassotoga saccharophila* gen. nov., sp. nov., isolated from an acidic terrestrial hot spring, and proposal of *Mesoaciditogales* ord. nov. and *Mesoaciditogaceae* fam. nov. in the phylum *Thermotogae*. Int J Syst Evol Microbiol 66:1045–1051. doi:10.1099/ijsem.0.000833. 26651491

[2] Reysenbach AL, Liu Y, Lindgren AR, Wagner ID, Sislak CD, Mets A, Schouten S 2013 *Mesoaciditoga lauensis* gen. nov., sp. nov., a moderately thermoacidophilic member of the order thermotogales from a deep-sea hydrothermal vent. Int J Syst Evol Microbiol 63:4724–4729. doi:10.1099/ijs.0.050518-0. 23959829

[3] Nelson KE, Clayton RA, Gill SR, Gwinn ML, Dodson RJ, Haft DH, Hickey EK, Peterson LD, Nelson WC, Ketchum KA, McDonald L, Utterback TR, Malek JA, Linher KD, Garrett MM, Stewart AM, Cotton MD, Pratt MS, Phillips CA, Richardson D, Heidelberg J, Sutton GG, Fleischmann RD, Eisen JA, White O, Salzberg SL, Smith HO, Venter JC, Fraser CM 1999 Evidence for lateral gene transfer between archaea and bacteria from genome sequence of *Thermotoga maritima*. Nature 399:323–329. doi:10.1038/20601. 10360571

[4] Zhaxybayeva O, Swithers KS, Lapierre P, Fournier GP, Bickhart DM, DeBoy RT, Nelson KE, Nesbo CL, Doolittle WF, Gogarten JP, Noll KM 2009 On the chimeric nature, thermophilic origin, and phylogenetic placement of the *Thermotogales*. Proc Natl Acad Sci U S A 106:5865–5870. doi:10.1073/pnas.0901260106. 19307556 PMC2667022

[5] Uritskiy GV, DiRuggiero J, Taylor J 2018 MetaWRAP: a flexible pipeline for genome-resolved metagenomic data analysis. Microbiome 6:158. doi:10.1186/s40168-018-0541-1. 30219103 PMC6138922

[6] De Coster W, D’Hert S, Schultz DT, Cruts M, Van Broeckhoven C 2018 NanoPack: visualizing and processing long-read sequencing data. Bioinformatics 34:2666–2669. doi:10.1093/bioinformatics/bty149. 29547981 PMC6061794

[7] Wick RR, Judd LM, Gorrie CL, Holt KE 2017 Unicycler: resolving bacterial genome assemblies from short and long sequencing reads. PLoS Comput Biol 13:e1005595. doi:10.1371/journal.pcbi.1005595. 28594827 PMC5481147

[8] Aziz R, Bartels D, Best A, DeJongh M, Disz T, Edwards R, Formsma K, Gerdes S, Glass E, Kubal M, Meyer F, Olsen G, Olson R, Osterman A, Overbeek R, McNeil L, Paarmann D, Paczian T, Parrello B, Pusch G, Reich C, Stevens R, Vassieva O, Vonstein V, Wilke A, Zagnitko O 2008 The RAST server: Rapid Annotations using Subsystems Technology. BMC Genomics 9:75. doi:10.1186/1471-2164-9-75. 18261238 PMC2265698

[9] Seemann T 2014 Prokka: rapid prokaryotic genome annotation. Bioinformatics 30:2068–2069. doi:10.1093/bioinformatics/btu153. 24642063

[10] Tanizawa Y, Fujisawa T, Nakamura Y 2018 DFAST: a flexible prokaryotic genome annotation pipeline for faster genome publication. Bioinformatics 34:1037–1039. doi:10.1093/bioinformatics/btx713. 29106469 PMC5860143

[11] Ogata H, Goto S, Sato K, Fujibuchi W, Bono H, Kanehisa M 1999 KEGG: Kyoto Encyclopedia of Genes and Genomes. Nucleic Acids Res 27:29–34. doi:10.1093/nar/27.1.29. 9847135 PMC148090

[12] Zdobnov EM, Apweiler R 2001 InterProScan: an integration platform for the signature-recognition methods in InterPro. Bioinformatics 17:847–848. doi:10.1093/bioinformatics/17.9.847. 11590104

[13] Huerta-Cepas J, Szklarczyk D, Forslund K, Cook H, Heller D, Walter MC, Rattei T, Mende DR, Sunagawa S, Kuhn M, Jensen LJ, von Mering C, Bork P 2016 eggNOG 4.5: a hierarchical orthology framework with improved functional annotations for eukaryotic, prokaryotic and viral sequences. Nucleic Acids Res 44:D286–D293. doi:10.1093/nar/gkv1248. 26582926 PMC4702882

